# Inflammasome proteins as biomarkers of traumatic brain injury

**DOI:** 10.1371/journal.pone.0210128

**Published:** 2018-12-31

**Authors:** Nadine Kerr, Stephanie W. Lee, Jon Perez-Barcena, Catalina Crespi, Javier Ibañez, M. Ross Bullock, W. Dalton Dietrich, Robert W. Keane, Juan Pablo de Rivero Vaccari

**Affiliations:** 1 Department of Neurological Surgery, Neuroscience Program, The Miami Project to Cure Paralysis, University of Miami Miller School of Medicine, Miami, FL, United States of America; 2 Department of Physiology and Biophysics, University of Miami Miller School of Medicine, Miami FL, United States of America; 3 Intensive Care Department, Son Espases Hospital, Palma de Mallorca, Spain; 4 Fundacio Institut d’Investigacio Sanitaria Illes Balears (IdISBa), Son Espases Hospital, Palma de Mallorca, Spain; 5 Department of Neurological Surgery, Son Espases Hospital, Palma de Mallorca, Spain; 6 Department of Neurological Surgery, The Miami Project to Cure Paralysis, University of Miami Miller School of Medicine, Miami, FL, United States of America; University of the Pacific, UNITED STATES

## Abstract

**Background:**

The inflammasome plays an important role in the inflammatory innate immune response after central nervous system (CNS) injury. Inhibition of the inflammasome after traumatic brain injury (TBI) results in improved outcomes by lowering the levels of caspase-1 and interleukin (IL)-1b. We have previously shown that inflammasome proteins are elevated in the cerebrospinal fluid (CSF) of patients with TBI and that higher levels of these proteins were consistent with poorer outcomes after TBI when compared to patients that presented these inflammasome proteins at lower levels.

**Methods and findings:**

Here we extend our work by analyzing serum from 21 TBI patients and CSF from 18 TBI patients compared to 120 serum samples and 30 CSF samples from no-TBI donor controls for the expression of caspase-1, apoptosis-associated speck-like protein containing a caspase recruitment domain (ASC), interleukin(IL)-1b and IL-18. Analysis was carried out using the Ella Simple Plex system (Protein Simple) to determine the sensitivity and specificity of inflammasome proteins as biomarkers of TBI. Receiver operator characteristic (ROC) curves, confidence intervals and likelihood ratios for each biomarker was determined. ROC curves, confidence intervals, sensitivity and specificity for each biomarker examined revealed that caspase-1 (0.93 area under the curve (AUC)) and ASC (0.90 AUC) in serum and ASC (1.0 AUC) and IL-18 (0.84 AUC) in CSF are promising biomarkers of TBI pathology. Importantly, higher protein levels (above 547.6 pg/ml) of ASC (0.91 AUC) were consistent with poorer outcomes after TBI as determined by the Glasgow Outcome Scale-Extended (GOSE).

**Conclusion:**

These findings indicate that inflammasome proteins are excellent diagnostic and predictive biomarkers of TBI.

## Introduction

A biomarker is a surrogate indicator of biological processes occurring in an individual. Biomarkers provide information about the pathology of a disease or condition, as well as the response to a pathogen or treatment. In the context of traumatic brain injury (TBI), biomarkers have the potential to be used as diagnostic markers of injury severity, as well as markers of response to treatment (monitoring biomarkers) or even as predictors of outcomes after trauma (predictive biomarkers).

Recently, the Brain Trauma Indicator has been authorized by the FDA to evaluate concussion in adults. This indicator is obtained by measuring the levels of ubiquitin C-terminal hydro-lase–L1 (UCH-L1) and glial acidic fibrillary protein (GFAP) in blood within 12 hours after trauma [1–3]. Other promising biomarkers have been suggested for diagnosis, monitoring and prognosis of traumatic brain injury (TBI) such as microtubule-associated protein-2 (MAP2, neuron-specific enolase (NSE), myelin basic protein (MBP), tau, s100β and neurofilament heavy chain protein (NF-H) [4–6]. However, these proteins are yet to be approved for the clinical care of TBI patients. In addition, heart fatty acid binding protein (H-FABP), when combined with interleukin (IL)-10, S100β and GFAP, has been shown to be elevated in patients positive for brain damage as determined by a head CT scan [7].

The innate immune response is a significant contributor to inflammation after TBI that is regulated by inflammasomes. To date several inflammasomes have been shown to play a role in the inflammatory response in the nervous system. For instance, the first inflammasome to be described in the nervous system was the NLRP1 inflammasome that is activated in neurons after spinal cord injury [8], brain injury [9] and stroke [10]. Moreover, the NLRP2 inflammasome in astrocytes has been shown to be involved in the inflammatory response after ATP stimulation [11]. The most studied inflammasome is the NLRP3 inflammasome and is is present in microglia and astrocytes [12–14]. Additionally, the NLRC4 inflammasome is present in astrocytes [14], and the AIM-2 inflammasome in neurons [15]. Inflammasome activation involves sensing of a particular trigger by cytosolic receptors such as NOD-like receptors (NLRs) or AIM-2 like receptors (ALRs). This activation process is triggered following the formation of large multiprotein complex called the inflammasome [16]. These inflammasome complexes undergo oligomerization that then serves as a platform to activate inflammatory caspases. However, some differences remain in that NLRP1 and NLRC4 can activate caspase-1 without ASC oligomerization, but ASC substantially improves the production of IL-1β. Thus, it is likely that inflammasomes that do not rely on ASC for assembly are less stable and as a result the inflammatory response mounted is weaker than it would be in the presence of ASC [17, 18]. Inflammasomes process inflammatory cytokines such as interleukin(IL)-1β and IL-18 through the activation of caspase-1 [18]. We have previously shown that inflammasomes contribute to the pathology of TBI and that inhibition of the inflammasome results in decreased inflammation and improved histopathological outcomes after brain injury [9]. Moreover, inflammasome proteins are secreted after injury in extracellular vesicles isolated from serum [19] and CSF [20] that add to the systemic inflammatory response after trauma [18, 21].

In this study, we provide receiver operator characteristic (ROC) curves with associated confidence intervals (CI) following analyses of serum and CSF samples from patients with TBI and from no-TBI control donors. In addition, we determine the sensitivity and specificity of inflammasome proteins to examine the potential of the inflammasome signaling proteins caspase-1, ASC, IL-1β and IL-18 as biomarkers of TBI.

## Methods

### Participants

Serum and CSF samples from patients with TBI and samples from no-TBI control donors were used in this study. Twenty-one TBI serum and 18 CSF samples were obtained after informed consent. Informed consent was obtained from a family member or proxy according to the University of Miami Miller School of Medicine IRB protocol # 20030154. All subjects were admitted to the Neurological Intensive Care Unit and/or the Ryder Trauma Center at Jackson Memorial Hospital (**Table 1**). Samples from normal donors (120 samples) were purchased from Bioreclamation*IVT*. The normal donor group consisted of samples obtained from 60 male and 60 female donors in the age range of 20 to 70 years old (**Table 2**). Thirty control CSF samples were obtained from Bioreclamation*IVT*. TBI Samples were collected three times a day for the first 5 days after patients arrived to the hospital. Samples were analyzed for the 1^st^, 2^nd^ collection (Day 1) as well as 4^th^ and 6^th^ collections (Day 2). Later time points were analyzed but the data was not shown to be significant (data not shown).

**Table 1:** Subjects with TBI.

Age	Gender	Race	Mechanism of Injury	Injury	GCS at resus	GOSE	Outcome
81 (Deceased)	Female	White	Blunt injury	Contusion, IVH, tSAH	3	1	Deceased
26	Male	Unknown	Gunshot wound	Contusion, tSAH,	3	3	Unfavorable
29	Male	Black	s/p MCC with helmet	Contusion, DAI	3	6	Favorable
69 (Deceased)	Male	Black	Blunt injury	SDH, tSAH	8	1	Deceased
23	Male	White	MVA w/blunt injury	SDH, t SAH	3	2	Unfavorable
22	Male	Black	GSW to back of head	LSDH, contusion	4	8	Favorable
70	Male	White	Blunt injury/assault	SDH	10	3	Unfavorable
21 (Deceased)	Male	White	Blunt injury pedestrian struck by car w/head injury	SDH	3	1	Deceased
22	Male	White	Blunt injury punched to head and fell	SDH	4	6	Moderate
80 (Deceased)	Male	White	Penetrating injury, GSW to posterior head	Contusion, IVH	3	1	Deceased
71	Female	White	Blunt injury. MVC		3	1	Deceased
51 (Deceased)	Male	Black	Blunt injury, hit by car while biking	tSAH, contusion, IVH	3	1	Deceased
39 (Deceased)	Male	Unknown	MVA w/ blunt injury	tSAH, IVH	3	1	Deceased
18	Female	White	Blunt injury	SDH, contusion	5	7	Favorable/Rehab
23 (Deceased)	Male	White	MCA w/blunt injury	Contusions, DAI, tSAH	3	1	Deceased
21	Male	Black	GSW to head w/ penetrating injury	tSAH, Contusion	5	4	Unfavorable
63	Male	White	Blunt injury	tSAH, SDH, Contusion	7	1	Deceased
21	Male	White	MVA w/blunt injury	Contusion, DAI, tSAH	5	3	Unfavorable
45	Male	White	MVC blunt injury	SDH, contusions, tSAH, IVH	8	2	Unfavorable
51	Male	White	Blunt injury	EDH, SDH, tSAH	3	7	Favorable/Rehab
22	Male	White	MVC w/blunt injury	tSAH, SDH, contusion	3	4	Unfavorable

**s/p**: status-post; **MCC**: Motor cycle crash; **MVA**: Motor Vehicle Accident; **GSW**: Gunshot wound; **MVC**: Motor Vehicle accident; **MCA**: Motor cycle accident; **IVH**: Intraventricular bleeding; **tSAH**: traumatic Subarachnoid bleeding; **DAI**: Diffuse Axonal Injury; **SDH**: Subdural hematoma; **LSDH**: Left Subdural hematoma; **GCS at resus**: Glasgow Coma Scale at resuscitation; **GOSE**: Glasgow Outcome Scale (Extended).

**Table 2:** Summary of control and subjects with TBI.

	Control	TBI
**Sample Size**	120	21
**Gender**		
Male	60	18
Female	60	3
**Age Range**	20–70 y/o	21–81 y/o
**Race**		
	White: 28	White: 13
	Black: 65	Black: 5
	Hispanic: 27	Unknown: 2
**ASC Levels**		
Range	105.6–458.3 pg/ml	138.5–1720 pg/ml
Mean	236.6 pg/ml	629 pg/ml
**Caspase-1 Levels**		
Range	0.952–2.703 pg/ml	0.918–20.55 pg/ml
a	1.436 pg/ml	4.540 pg/ml
**IL-18 Levels**		
Range	40.5–422.7 pg/ml	43.72–587.7 pg/ml
Mean	213.5 pg/ml	218.625 pg/ml
**IL-1beta Levels**		
Range	0.413–3.276 pg/ml	0.4–4.674 pg/ml
Mean	1.214 pg/ml	1.0618 pg/ml

**y/o**: years old.

### Simple Plex Assay

Analysis of inflammasome protein concentration in serum and CSF samples were performed using the Ella System (Protein System) as described in [22, 23].

### Biomarker analyses

Data obtained by the Simple Plex assay were analyzed with Prism 7 software (GraphPad). Analyses were carried after removing outliers, and the area under the ROC curve and the 95% confidence interval (CI) were determined. The p-value of significance used was <0.05. Sensitivity and specificity of each biomarker was obtained for a range of different cut-off points. Samples that resulted in values outside the level of detection of the assay were not included in the analyses [22, 23]. Multiple group comparisons were carried out using a Kruskal-Wallis test ANOVA followed by Dunn’s multiple comparisons test. Paired comparisons were performed using a two-tailed t-test. Normality was evaluated using the D'Agostino and Pearson normality test. Difference in sample size throughout the study is due to the removal of outliers and because the protein concentration of some samples was out of the range of detection of the assay for some of the analytes and was not evaluated. The box plot shows the 5^th^ and 95^th^ percentiles, and the line inside the box represents the median.

## Results

### ASC and caspase-1 are elevated in the serum of patients after TBI

**Fig 1:**
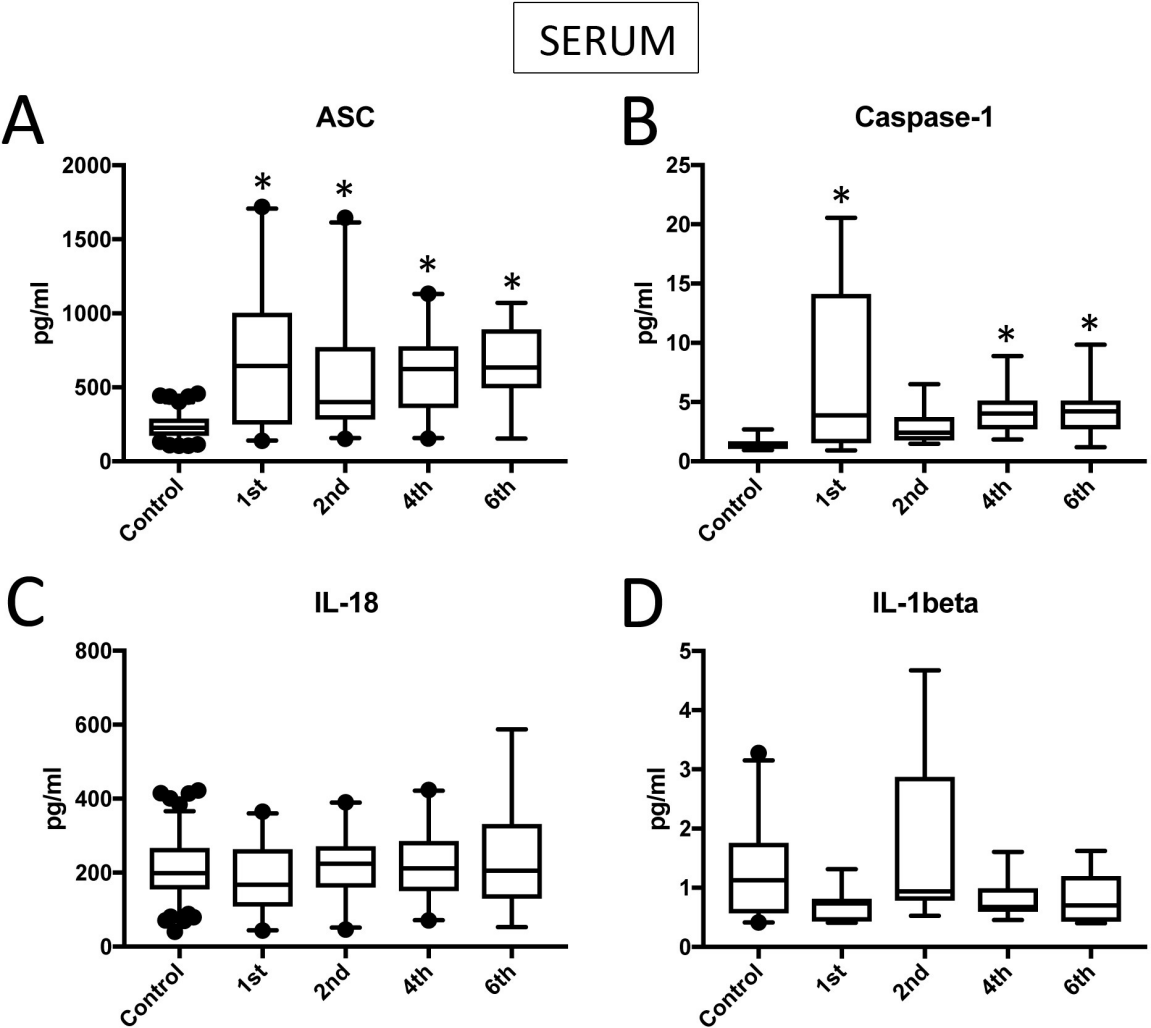
Inflammasome proteins are elevated in the serum of TBI patients. Protein levels in pg/ml of ASC (**A**), caspase-1 (B), IL-18 (C) and IL-1β (D) in serum samples from patients with TBI and healthy donors (controls). ASC: N = 120 control, 20 TBI. Caspase-1: N = 11 control, 19 TBI. IL-18: N = 120 control, 21 TBI. IL-1β: N = 25 control, 10 TBI. Box and whiskers are shown for the 5^th^ and 95^th^ percentile. * p < 0.05.

We analyzed serum samples (**S1 Table**) from TBI patients and compared them to serum from healthy/control individuals using a Simple Plex assay (Protein Simple) for the protein expression levels of ASC (**Fig 1A**), caspase-1 (**Fig 1B)**, IL-18 (**Fig 1C)** and IL-1β (**Fig 1D**). Samples were collected three times a day for five days. Data shown corresponds to the 1^st^, 2^nd^ 4^th^ and 6^th^ collection. We determined that the protein levels of ASC were higher in TBI samples (1^st^ collection: 693.5+/-108.5 pg/ml, 2^nd^: 562.6+/-84.51 pg/ml, 4^th^: 601.7+/-64.4 pg/ml, 6^th^: 658.2+/-79.69 pg/ml) when compared to samples from subjects without TBI (control, 236.6+/-7.758 pg/ml) (**Fig 1A**). Thus, indicating that inflammasome proteins are elevated in serum after TBI.

### ASC and Caspase-1 are promising serum biomarkers of TBI

**Fig 2:**
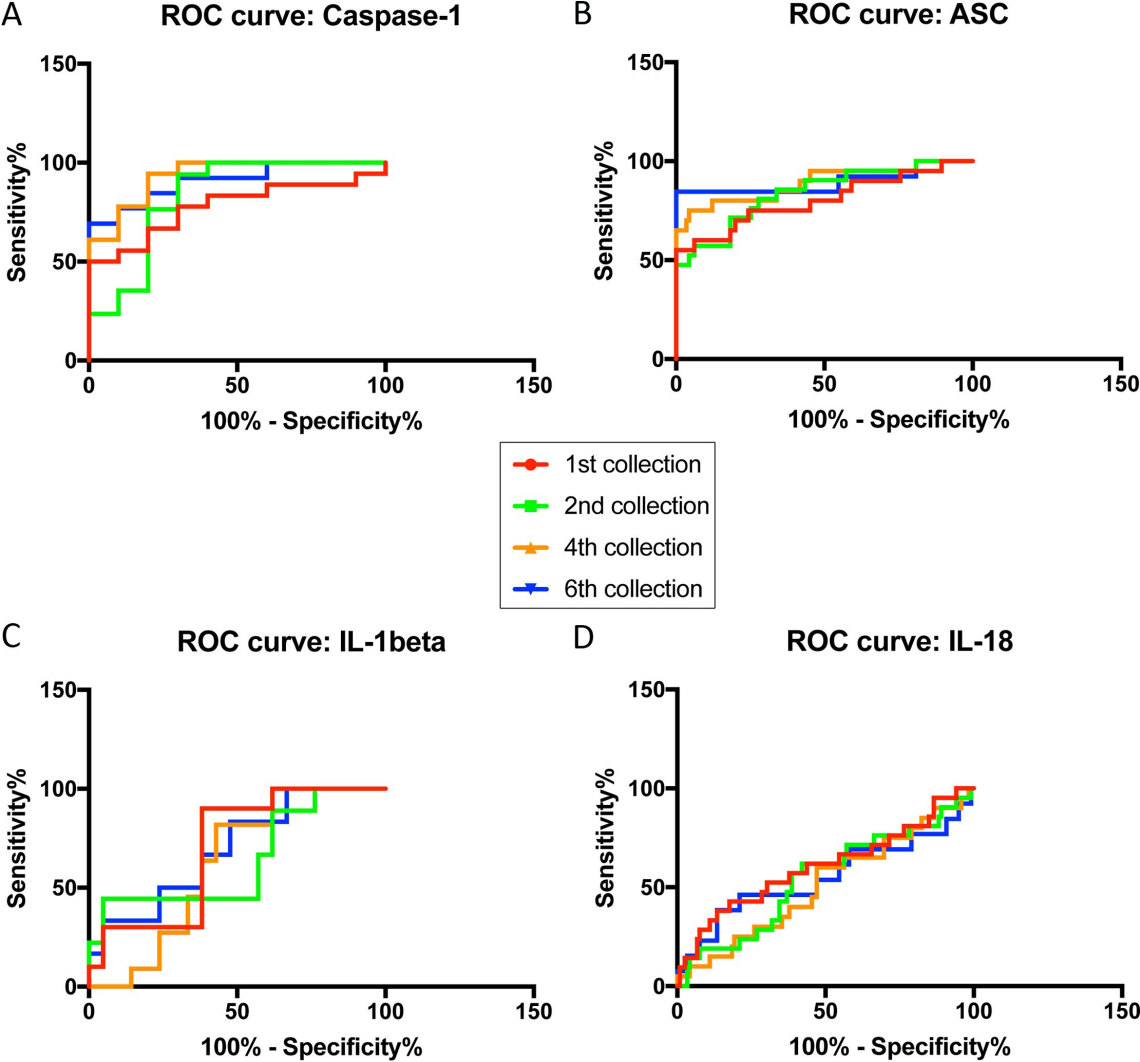
ROC curves for caspase-1 (**A**), ASC (**B**), IL-1β (**C**) and IL-18 (**D**) from serum samples of TBI patients and healthy donors.

To determine if these inflammasome signaling proteins are reliable biomarkers for TBI in serum, we determined the area under the curve (AUC) for caspase-1 (**Fig 2A**), ASC (**Fig 2B**), IL-1β (**Fig 2C**) and IL-18 (**Fig 2D**). AUC values were highest for caspase-1 and ASC (**Fig 2**) with 0.93 (4^th^ collection) and 0.90 (6^th^ collection), respectively (**Table 3**).

**Table 3:** ROC analysis results for inflammasome signaling proteins in serum including area, standard error (STD. ERROR), 95% confidence interval (CI) and p-value for collections 1^st^, 2^nd^, 4^th^ and 6^th^.

Caspase-1 Serum	AREA	STD. ERROR	95% C.I.	P VALUE
1^st^ Collection	0.78	0.08772	0.6058 to 0.9497	0.01
2^nd^ Collection	0.83	0.0479	0.8395 to 1.027	0.005
4^th^ Collection	0.93	0.1407	0.8353 to 0.8869	0.0002
6^th^ Collection	0.91	0.06065	0.7888 to 1.027	0.001
**ASC Serum**				
1^st^ Collection	0.80	0.06472	0.6762 to 0.9299	<0.0001
2^nd^ Collection	0.84	0.05026	0.7425 to 0.9395	<0.0001
4^th^ Collection	0.89	0.04898	0.7931 to 0.9851	<0.0001
6^th^ Collection	0.90	0.0697	0.759 to 1.032	<0.0001
**IL-1beta Serum**				
1^st^ Collection	0.7	0.0965	0.5109 to 0.8891	0.0759
2^nd^ Collection	0.64	0.1182	0.4085 to 0.8719	0.2304
4^th^ Collection	0.6234	0.09765	0.432 to 0.8148	0.2582
6^th^ Collection	0.6984	0.1162	0.4707 to 0.9261	0.1448
**IL-18 Serum**				
1^st^ Collection	0.61	0.07475	0.4593 to 0.7524	0.1227
2^nd^ Collection	0.55	0.07064	0.4082 to 0.6851	0.4966
4^th^ Collection	0.51	0.0713	0.372 to 0.6515	0.8666
6^th^ Collection	0.55	0.1015	0.3532 to 0.7509	0.5387

Furthermore, the cut-off point for caspase-1 was 1.943 pg/ml with 94% sensitivity and 89% specificity (**Table 4**). For ASC, the cut-off point was 451.3 pg/ml with 85% sensitivity and 99% specificity (**Table 4**); whereas values for caspase-1 showed 100% sensitivity and the cut-off point was 1.679 pg/ml with 77.78% specificity. For ASC, the cut-off point was 153.4 pg/ml and a 19% specificity. In the case of caspase-1, for 100% specificity, the cut-off point was 2.717 pg/ml with 78% sensitivity. For ASC with 100% specificity, the cut-off point was 462.4 pg/ml with 85% sensitivity. Thus, these findings indicate that caspase-1 and ASC are reliable serum biomarkers for TBI.

**Table 4:** ROC analysis results for caspase-1 and ASC in serum including cut-off point in pg/ml, sensitivity and specificity, as well as positive and negative likelihood ratios (LR+/LR-).

Caspase-1 Serum	Cut-off point (pg/ml)	Sensitivity (%)	Specificity (%)	LR +	LR -
1^st^ Collection	> 1.439	83	67	2.50	0.25
2^nd^ Collection	> 1.531	94	78	4.24	0.08
4^th^ Collection	> 1.943	94	89	8.50	*0*.*06*
6^th^ Collection	> 1.947	85	89	7.62	0.17
**ASC Serum**					
1^st^ Collection	> 210	85	43	1.50	0.35
2^nd^ Collection	> 275	81	72	2.91	0.26
4^th^ Collection	> 339.4	80	88	6.57	0.23
6^th^ Collection	> 451.3	85	99	97.26	0.16

### ASC and IL-18 are elevated in the CSF of patients after TBI

**Fig 3:**
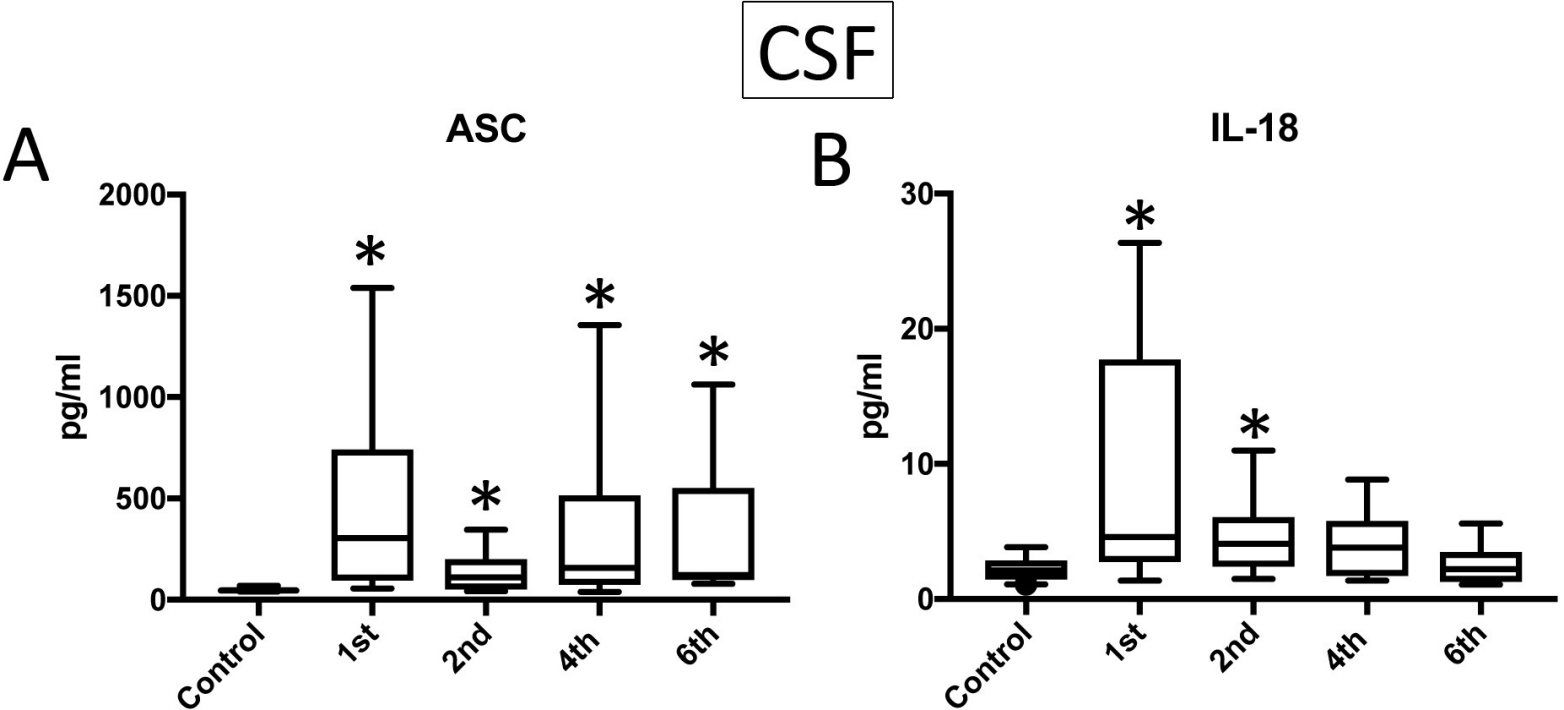
Inflammasome proteins are elevated in the CSF of TBI patients. Protein levels in pg/ml of ASC (**A**) and IL-18 (**B**) in CSF samples from patients with TBI and healthy donors (controls). ASC: N = 21 control, 15 TBI. IL-18: N = 24 control, 16 TBI. Box and whiskers are shown for the 5^th^ and 95^th^ percentile. * p < 0.05.

We then analyzed CSF samples (**S2 Table**) from TBI patients and compared them to CSF from no-TBI control individuals for the protein expression of ASC (Fig 3A) and IL-18 (Fig 3B). The protein levels of ASC and IL-18 were higher in TBI samples (ASC 1^st^: 473.8+/-129.4 pg/ml, 2^nd^: 132.8+/-24.45 pg/ml, 4^th^: 362.1+/-106.3 pg/ml, 6^th^: 325.4+/-124.4 pg/ml, IL-18: 1^st^: 9.164+/-2.169, 2^nd^: 4.626+/-0.7595 pg/ml, 4^th^: 4.169+/-0.7801 pg/ml) when compared to control samples (ASC: 48.52+/-2.259 pg/ml, IL-18: 2.246+/-0.1971). Moreover, the protein concentration of caspase-1 and IL-1β in the CSF was below the level of detection of the assay. Thus, we were unable to carry out any statistical analysis for these two proteins in the present study. These findings are consistent with previous reports indicating a role for the inflammasome in the pathology of TBI [20, 24].

### ASC and IL-18 are reliable CSF biomarkers of TBI

**Fig 4:**
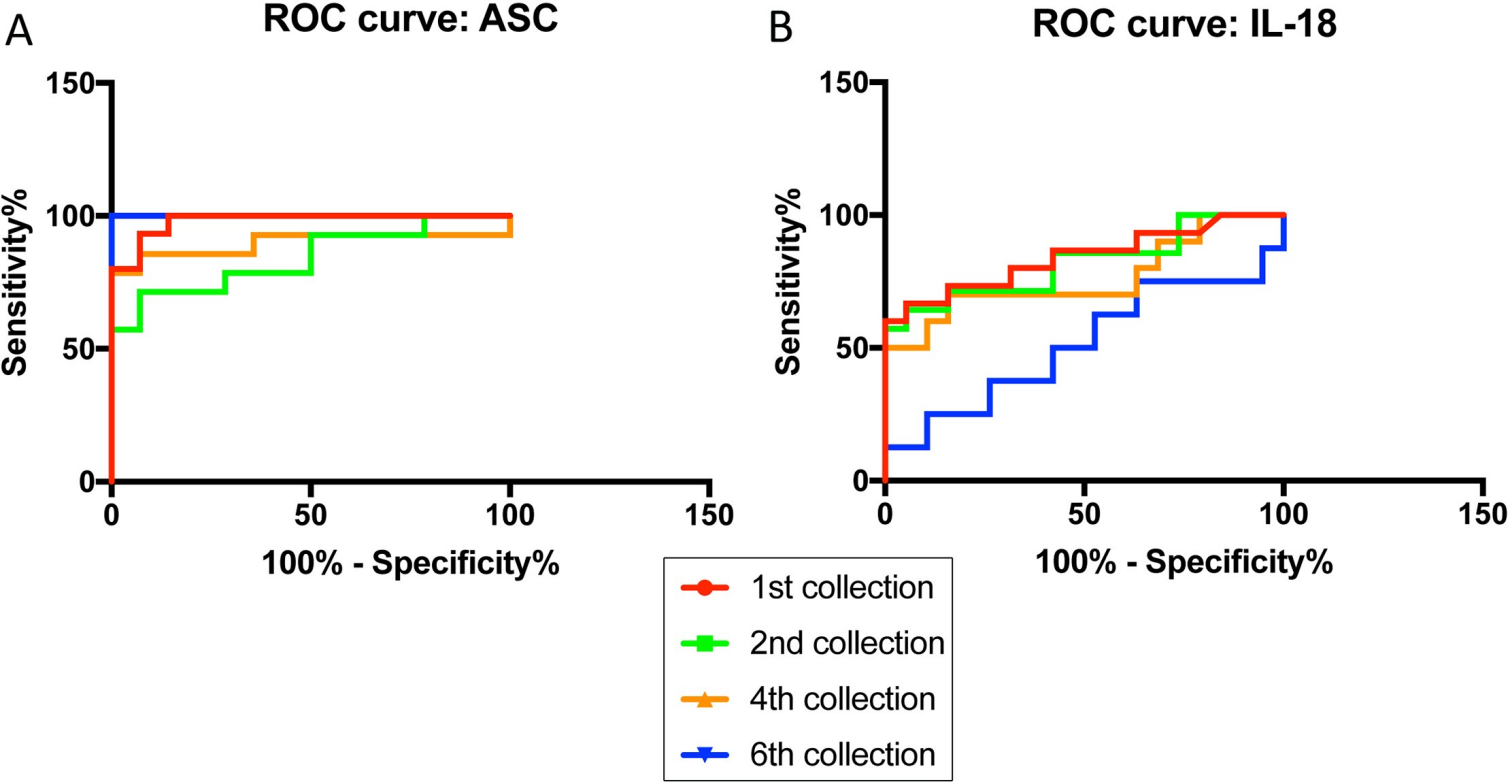
ROC curves for ASC (**A**) and IL-18 (**B**) from CSF samples of TBI patients and healthy donors.

Next, we determined whether ASC and IL-18 are reliable biomarkers in CSF for TBI. The area under the curve (AUC) for ASC (**Fig 4A**) was 1.0 for the 6^th^ collection (**Table 5**), and for IL-18 (**Fig 4B**) the AUC was 0.84 for the 1^st^ collection (**Table 5**), indicating that ASC is a more reliable biomarker than IL-18 in CSF after TBI.

**Table 5:** ROC analysis results for ASC and IL-18 in CSF including area, standard error (STD. ERROR), 95% confidence interval (CI) and p-value for collections 1^st^, 2^nd^, 4^th^ and 6^th^.

ASC CSF	AREA	STD. ERROR	95% C.I.	P VALUE
1^st^ Collection	0.981	0.0195	0.9427 to 1.019	<0.0001
2^nd^ Collection	0.8418	0.07661	0.6917 to 0.992	0.0021
4^th^ Collection	0.898	0.07262	0.7556 to 1.04	0.0003
6^th^ Collection	1	0	1 to 1	0.0001
**IL-18 CSF**				
1^st^ Collection	0.8404	0.0731	0.6971 to 0.9836	0.0008
2^nd^ Collection	0.8195	0.07969	0.6634 to 0.9757	0.002
4^th^ Collection	0.7632	0.1061	0.552 to 0.9711	0.9711
6^th^ Collection	0.5132	0.1344	0.2498 to 0.7765	0.9154

The cut-off point for ASC was 74.33 pg/ml with 100% sensitivity and 100% specificity (**Table 6**). For IL-18, the cut-off point was 2.722 pg/ml with 80% sensitivity and 68% specificity (**Table 6**). In the case of IL-18, for 100% specificity, the cut-off point was 3.879 pg/ml with 60% sensitivity, and for 100% sensitivity, the cut-off point was 1.358 pg/ml with 16% specificity. Thus, these findings indicate that ASC and IL-18 are promising biomarkers in CSF for TBI.

**Table 6:** ROC analysis results for ASC and IL-18 in CSF including cut-off point in pg/ml, sensitivity and specificity, as well as positive and negative likelihood ratios (LR+/LR-).

ASC CSF	Cut-off point (pg/ml)	Sensitivity (%)	Specificity (%)	LR +	LR -
1^st^ Collection	> 55.11	100	85.71	7	0
2^nd^ Collection	> 50.25	78.57	64.29	2.20	0.33
4^th^ Collection	> 64.58	85.71	92.86	12	0.15
6^th^ Collection	> 74.33	100	100		0
**IL-18 CSF**					
1^st^ Collection	> 2.722	80	68.42	2.53	0.29
2^nd^ Collection	> 2.221	85.71	57.89	2.04	0.25
4^th^ Collection	> 3.055	70	84.21	4.43	0.36
6^th^ Collection	> 1.707	75	36.84	1.19	0.68

### ASC is elevated in the serum of patients with unfavorable outcomes after TBI

**Fig 5:**
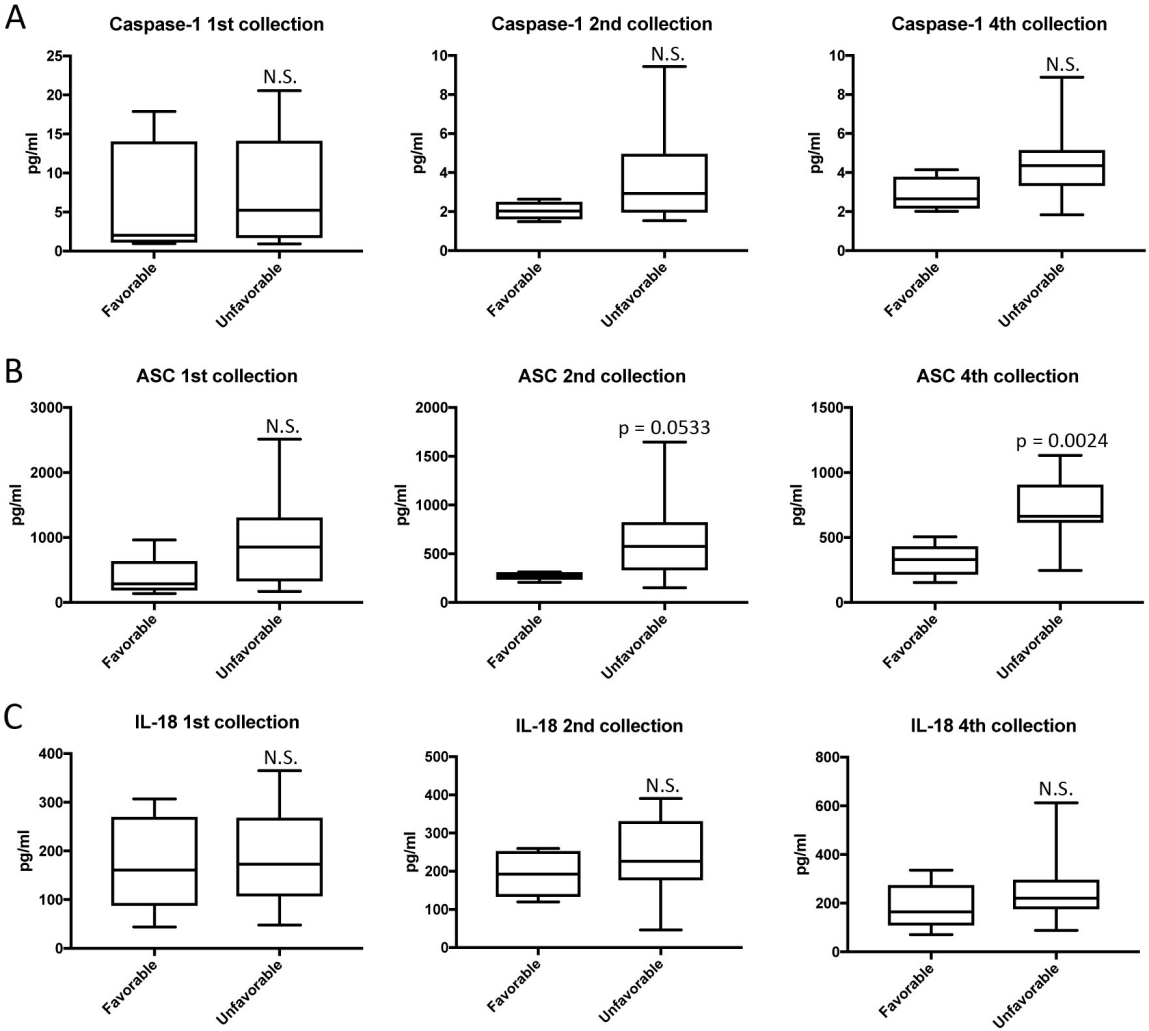
Inflammasome proteins as predictive biomarkers of TBI. Protein levels in pg/ml of caspase-1 (**A**), ASC (**B**), and IL-18 (**C**) in serum samples from patients with TBI. Groups were divided into favorable and unfavorable outcomes based on the GOSE. p-value of significance is shown above each box plot. Box and whiskers are shown for the 5^th^ and 95^th^ percentile. Caspase-1: N = 4 favorable and 16 unfavorable ASC: N = 5 favorable and 16 unfavorable; and IL-18: N = 5 favorable and 16 unfavorable.

We then separated the TBI patients according to their clinical outcomes (**S3 Table**); either favorable or unfavorable outcomes based on the Glasgow Outcome Scale-Extended (GOSE) in which patients with a score of 6 to 8 were considered to have favorable outcomes and those with a score of 1 to 4 were considered to have unfavorable outcomes (**Table 1**). We found that the protein level of ASC was higher in the serum of TBI patients with unfavorable outcomes (2^nd^: 652.9+/-100.9pg/ml, 4^th^: 719.2+/-68.14 pg/ml) when compared to the samples obtained from patients with favorable outcomes (2^nd^: 273.7+/-19.41 pg/ml, 4^th^: 327.6+/-53.84 pg/ml) (**Fig 5B**), whereas the caspase-1 (**Fig 5A**) and IL-18 (**Fig 5C**) levels were not statistically different between the two groups.

### ASC is a promising predictive biomarker of TBI in serum

**Fig 6:**
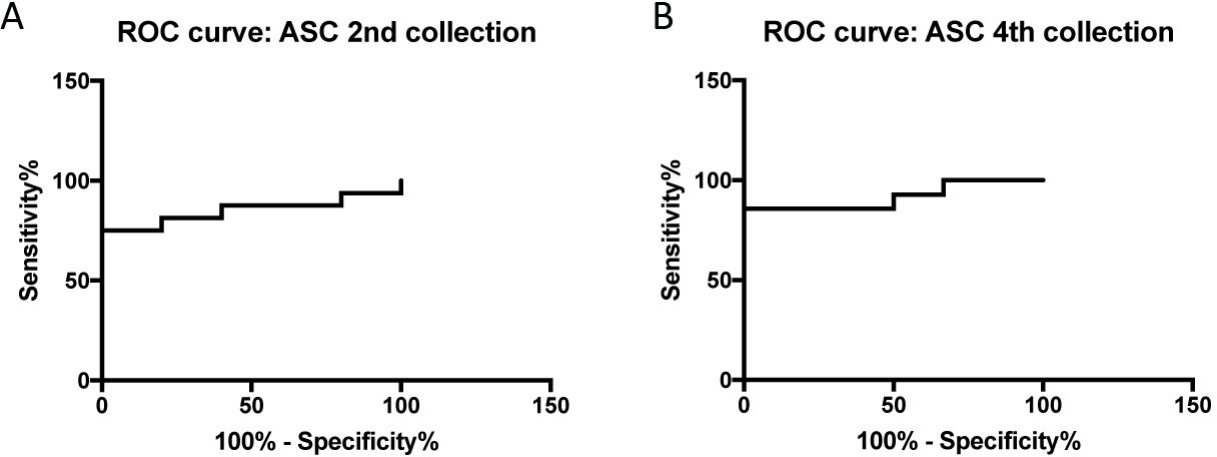
ROC curves for ASC outcomes (Favorable vs. Unfavorable) for the 2^nd^ and 4^th^ collection.

To determine if ASC in serum can be used as predictive biomarkers of TBI, we determined the AUC for ASC at the 2^nd^ (**Fig 6A**) and 4^th^ collection (**Fig 6B**). The AUC for ASC was 0.9167 in the 4^th^ collection with a CI between 0.7914 and 1.042 (**Table 7**). Furthermore, the cut-off point was 547.6 pg/ml with 86% sensitivity and 100% specificity (**Table 8)**. Thus, these findings indicate that ASC is an excellent predictive biomarker of TBI.

**Table 7:** ROC analysis results for ASC in serum for Favorable vs Unfavorable outcomes, including area, standard error (STD. ERROR), 95% confidence interval (CI) and p-value for collections 1^st^, 2^nd^ and 4^th^.

ASC Serum (GOSE)	AREA	STD. ERROR	95% C.I.	P VALUE
1^st^ Collection	0.7625	0.1133	0.544 to 0.9846	0.0829
2^nd^ Collection	0.85	0.08355	0.6862 to 1.014	0.0208
4^th^ Collection	0.9167	0.06391	0.7914 to 1.042	0.0039

**Table 8:** ROC analysis results for ASC in serum for Favorable vs Unfavorable outcomes, including cut-off point in pg/ml, sensitivity and specificity, as well as positive and negative likelihood ratios (LR+/LR-) for collections 1^st^, 2^nd^ and 4^th^.

ASC Serum (GOSE)	CUT-OFF POINT (pg/ml)	SENSITIVITY (%)	SPECIFICITY (%)	LR +	LR -
1^st^ Collection	> 353.7	75	80	3.75	0.31
2^nd^ Collection	> 311.2	81.25	80	4.06	0.23
4^th^ Collection	> 547.6	85.71	100		0.14

## Discussion

TBI affects approximately 10 million people worldwide and has become a major cause of death and disability [25]. TBI severity is categorized as mild, moderate or severe. Signs and symptoms of TBI may include loss of consciousness, amnesia, nausea, dizziness, headaches, cognitive decline, structural brain damage and presence of other neurological symptoms [26]. The level of consciousness after TBI is often used to determine the severity of trauma in the Glasgow Coma Scale (GCS) score. This score is combined with brain radiographic imaging, other neurological findings and fluid biomarkers to assess the clinical status of the patient[27]. Fluid biomarkers can be obtained from CSF, serum or plasma. Potential biomarkers for the care of patients with TBI include total tau (T-Tau), amyloid-β (Aβ), UCH-L1, NSE, spectrin-α chain, S100B and GFAP [26]. Although there appears to be a variety of candidate proteins that may be potentially used as biomarkers, many studies have only looked at protein levels across patient cohorts including controls. Many of the studies aiming at identify biomarkers of TBI have failed to use the adequate statistical analyses that are needed to determine the suitability of a protein as a biomarker. These analyses include determination of the AUC, sensitivity, specificity and likelihood ratio as well as positive and negative predictive values [23]. Currently, there is only one approved FDA test for blood-based biomarkers in the United States, involving GFAP and UCH-L1, [28] which was approved this year.

In the present study, we show that ASC and IL-18 are reliable biomarkers for TBI in CSF with AUC values of 1.0 and 0.84, respectively. Most importantly, since obtaining CSF is a very invasive procedure, our finding that inflammasome proteins are elevated in serum, opens the possibility of using a less invasive test such as a blood test for biomarker analysis. In serum, we found that the AUC values for ASC was 0.90 and for caspase-1, 0.93. Thus, of the inflammasome proteins that we tested in this study, caspase-1 and ASC are the best candidate biomarkers in serum for the care of patients with brain injury. Thus, the development of a clinically validated brain biomarker test involving inflammasome proteins has the potential to alter diagnostic approaches and development of clinical care procedures of TBI subjects.

The importance of inflammasome regulation in TBI secondary injury processes has been recently reported. The NLRP1 inflammasome plays a significant role in the inflammatory response after TBI[9], and inhibition of NLRP1 signaling by antibody neurtralization results in decreased inflammation and improved histopathology after brain injury [9]. Additionally, the NLRP3 inflammasome is activated after brain injury [29, 30], and inhibition of the NLRP3 inflammasome results in decreased inflammation and improved neurological function in a mouse model of brain injury [31, 32]. The AIM2 inflammasome has been associated with the inflammasome-mediated cell death mechanism of pyroptosis, thus affecting blood brain barrier permeability after trauma [15, 33]. Inflammasome proteins are elevated in the CSF of patients with TBI, and high levels of these proteins correlate with poor outcomes in the chronic stages after injury [24]. Lastly, hyperbaric oxygen therapy, [34] hypothermia, [35] omega-3 fatty acids, [36] resveratrol[37] and propofol [38] provide beneficial effects after TBI by lowering inflammasome activation. Thus, innate immune inflammatory processes regulated by inflammasomes play an integral role in pathological processes following TBI.

Standards for Reporting Diagnostic accuracy studies (STARD) guidelines were used in this study. However, our study has several limitations. For example, the sample collection time points from patients after trauma was not uniform. As a result, the second collection of samples from patients varied after trauma. In addition, we were unable to collect CSF from all patients, and as a result the number of CSF samples are lower when compared to the number of serum samples. In addition, most samples collected in this study were obtained from subjects with severe TBI. Thus, future studies are needed to evaluate the prognostic potential of these biomarker in patients with mild TBI. Moreover, patients enrolled in this study suffered from isolated head trauma (i.e. gunshot wound) or had polytrauma (i.e. motorvehicle accident). As a result, it is likely that the effects of trauma on serum levels of inflammasome proteins are not entirely related to brain injury but to tissue damage originating in other organs such as lung [19], spleen or liver. Lastly, our study involved Whites and Blacks but did not contain Hispanics or other races in the TBI cohort.

We have recently shown that inflammasome proteins may be used as biomarkers of multiple sclerosis, stroke and depression [23, 39, 40]. However, patients in each disease category present different levels of inflammasome proteins and these levels may vary depending on disease severity. Thus, it is likely that these proteins may be used to monitor disease inflammatory progression or severity. The specificity of inflammasome proteins as biomarkers may be altered by changing the cut-off point to reflect a different sensitivity since sensitivity and specificity are inversely correlated such that as specificity increases, sensitivity decreases. In this study, we chose to set cut-off points that provided high sensitivity and the highest possible specificity for all analytes. Therefore, for clinical purposes the sensitivity should be set at the highest level and the specificity may be increased by the addition of other clinical findings such as imaging biomarkers, symptoms or other fluid-based biomarkers.

In conclusion, in this study we show that the inflammasome protein ASC may be use as a biomarker of TBI in serum and CSF, whereas caspase-1 may be used as a serum biomarker and IL-18 as a biomarker in CSF. In future studies, we will analyze these biomarkers in larger cohorts of patients with more stratified and specific types of brain injury. Importantly, the identification of these proteins as biomarkers is also indicative of their potential as therapeutic targets for the treatment of inflammation after trauma.

## Supporting information

S1 Table Raw data used in the analysis of biomarkers in serum samples. (PDF)

S2 Table Raw data used in the analysis of biomarkers in CSF samples. (PDF)

S3 Table Raw data used in the analysis of biomarkers in serum samples for patients with favorable vs unfavorable outcomes. (PDF)
