# Supplementary material for: Inflammasome proteins as biomarkers of traumatic brain injury
Source: PLoS One. 2018 Dec 31;13(12):e0210128. doi: 10.1371/journal.pone.0210128 (PMC6312377; doi:10.1371/journal.pone.0210128)
Supplement: S1 Table — (PDF) [file pone.0210128.s001.pdf]

SERUM RAW DATA

ASC Serum

Control	1st	2nd	4th	6th
166.422	515.474	571.262	784.492	633.406
359.219	809.502	774.894	872.346	567.095
331.030	289.049	276.968	262.031	605.812
152.194	395.333	243.882	347.862	634.469
250.470	199.004	312.580	646.659	893.074
273.621	138.527	309.768	400.377	154.626
216.652	210.476	465.337	626.330	1070.857
272.456	171.112	288.089	647.329	523.192
204.526	234.834	260.629	154.042	839.426
350.174	771.691	385.558	621.237	466.500
132.228	1006.319	769.957	1016.983	1059.773
143.440	900.333	581.385	679.974	892.199
297.539	1719.545	1646.571	234.110	215.866
219.284	312.122	313.869	753.431	
243.639	1054.776	726.874	1104.345	
250.541	997.525	840.706	246.274	
302.363	1485.805	152.318	1132.170	
226.785	302.821	969.973	589.010	
272.186	1392.648	1315.083	409.038	
233.228	963.914	207.074	506.148	
132.603		401.481		
444.319				
145.821				
141.930				
105.624				
151.473				
135.672				
146.659				
161.523				
186.155				
133.385				
135.247				
136.384				
395.447				
135.859				
106.029				
135.105				
320.841				
269.530				
109.826				

230.161
249.906
283.705
182.408
185.199
322.904
264.572
298.507
209.917
289.940
171.779
303.014
282.175
151.356
305.075
351.962
272.979
211.962
232.009
177.081
437.708
184.625
244.354
191.421
288.621
398.377
199.762
252.485
181.703
330.935
190.824
210.148
170.652
458.290
197.636
196.394
196.853
436.778
385.048
354.182
231.457
315.760
250.861
252.414

172.535
149.351
192.292
382.388
184.099
233.735
402.492
216.962
221.490
227.473
326.491
245.108
156.376
148.367
262.040
143.170
174.188
242.634
169.648
259.639
180.001
114.277
186.943
290.913
256.004
325.877
159.983
193.932
381.314
284.047
258.402

Caspase-1 Serum

Control	1st	2nd	4th	6th
0.952	2.052	3.328	4.649	3.375
1.016	1.544	2.522	3.455	2.025
1.399	1.478	1.945	2.583	6.761
1.080	2.960	1.588	1.836	4.211
1.277	0.970	1.540	3.989	5.722
1.869	1.733	2.119	2.731	1.194
1.522	0.918	3.339	5.130	9.843
2.703	4.818	2.062	5.148	3.772
1.102	7.260	5.962	3.904	4.301
	7.985	2.422	5.165	3.656

14.771	2.266	2.570	4.508
2.567	2.637	2.016	4.565
14.031	4.123	4.065	1.780
14.411	1.588	2.892	
1.158	6.516	8.889	
20.554	1.486	6.015	
17.892	4.639	4.145	
5.647		4.770	

IL-18 Serum

Control	1st	2nd	4th	6th
170.351	318.533	221.660	260.689	283.761
160.553	104.232	95.383	123.252	94.196
121.050	233.355	259.711	335.670	396.293
214.514	47.804	46.586	130.532	116.643
188.606	104.986	228.044	213.299	316.114
153.321	43.723	119.592	145.726	53.049
146.399	119.067	237.482	227.538	347.281
288.921	77.442	224.073	210.336	185.591
248.157	130.457	146.893	70.752	307.239
169.554	167.342	120.714	168.783	142.393
239.034	304.151	384.541	293.650	587.682
366.142	213.145	171.978	193.186	205.570
168.911	252.959	347.315	377.697	193.854
95.077	160.849	192.685	212.354	
362.969	178.019	215.263	230.724	
160.703	364.816	390.554	207.186	
174.991	190.116	189.629	297.998	
127.225	111.408	284.101	88.031	
299.546	125.183	233.669	164.056	
298.910	306.775	246.165	423.688	
69.956	273.831	369.261		
120.756				
112.281				
337.715				
128.537				
214.499				
126.888				
133.854				
117.710				
133.938				
119.891				

112.044
172.644
177.269
109.077
190.010
88.034
339.309
199.045
79.359
301.794
201.729
220.095
176.761
414.275
365.247
250.060
422.656
154.709
340.058
401.193
197.874
172.644
296.602
71.083
259.665
161.044
264.877
181.314
267.299
254.793
168.910
215.852
322.212
352.547
270.429
214.651
137.754
157.465
225.436
331.824
221.249
182.040
230.803
141.579

193.753
228.834
249.756
196.698
249.668
216.769
227.780
178.831
280.246
260.112
326.348
127.739
277.242
198.420
81.272
40.500
304.396
162.690
384.035
180.761
125.331
213.080
195.529
178.879
213.791
242.073
152.610
101.294
306.446
200.099
301.409
117.147
155.932
212.702
240.876
193.350
196.446
275.584
154.959
228.121
202.907
287.210
343.337
415.221

<u>IL-1β Serum</u>

Control	1st	2nd	4th	6th
1.128	0.431	3.291	0.625	0.400
1.770	0.760	0.846	0.994	1.054
0.484	0.709	0.526	0.592	1.622
0.620	0.805	0.913	0.457	0.583
0.453	0.410	2.360	1.507	0.434
0.444	0.849	2.452	0.754	0.817
0.413	1.315	0.939	1.606	
1.629	0.802	4.674	0.950	
1.305	0.427	0.715	0.647	
1.135	0.714		0.540	
1.607			0.675	
1.867				
2.028				
0.522				
3.276				
1.021				
0.648				
0.875				
1.747				
1.897				
0.617				
