# Supplementary material for: Inflammasome proteins as biomarkers of traumatic brain injury
Source: PLoS One. 2018 Dec 31;13(12):e0210128. doi: 10.1371/journal.pone.0210128 (PMC6312377; doi:10.1371/journal.pone.0210128)
Supplement: S2 Table — (PDF) [file pone.0210128.s002.pdf]

CSF RAW DATA

ASC CSF

Control	1st	2nd	4th	6th
69.363	303.430	218.119	127.923	116.442
43.319	396.648	226.564	619.151	599.695
52.654	64.690	177.206	473.175	118.092
40.637	62.767	52.413	38.678	126.946
42.854	98.833	44.082	481.385	90.198
48.779	502.166	195.957	76.782	79.288
42.571	1425.894	42.393	988.668	1062.003
60.266	113.010	62.556	68.889	410.139
40.626	56.335	44.651	389.402	
53.879	578.210	345.010	50.113	
44.047	1018.515	68.063	122.328	
51.714	1539.883	94.809	1355.021	
40.705	741.984	125.809	90.967	
47.799	93.689	162.107	186.440	
	111.380			

IL-18 CSF

Control	1st	2nd	4th	6th
1.089	7.060	7.841	3.536	3.699
1.244	9.796	9.053	8.833	1.063
1.365	3.917	4.026	1.573	1.751
1.942	2.262	2.327	1.763	2.838
2.709	1.365	4.379	3.231	2.446
3.723	2.734	1.517	7.304	1.996
2.851	10.828	2.417	1.366	1.101
1.446	4.591	10.986	4.695	5.591
2.105	1.810	1.482	4.101	
1.663	17.747	4.151	5.284	
2.686	26.372	2.982		
3.841	22.916	3.732		
1.552	19.285	5.457		
2.782	3.771	4.414		
2.879	3.010			
2.114				
1.351				
1.878				
3.449				
