# Supplementary material for: Inflammasome proteins as biomarkers of traumatic brain injury
Source: PLoS One. 2018 Dec 31;13(12):e0210128. doi: 10.1371/journal.pone.0210128 (PMC6312377; doi:10.1371/journal.pone.0210128)
Supplement: S3 Table — (PDF) [file pone.0210128.s003.pdf]

FAVORABLE VS UNFAVORABLE

ASC 1st collection

Favorable	Unfavorable
289.049	515.474
138.527	809.502
234.834	395.333
312.122	199.004
963.914	210.476
	171.112
	771.691
	1006.319
	900.333
	1719.545
	1054.776
	997.525
	1485.805
	302.821
	1392.648
	2513.009

ASC 2nd collection

Favorable	Unfavorable
276.968	571.262
309.768	774.894
260.629	243.882
313.869	312.580
207.074	465.337
	288.089
	385.558
	769.957
	581.385
	1646.571
	726.874
	840.706
	152.318
	969.973
	1315.083
	401.481

ASC 4th collection

Favorable	Unfavorable
262.031	784.492
400.377	872.346

154.042	347.862
234.110	646.659
409.038	626.330
506.148	647.329
	621.237
	1016.983
	679.974
	753.431
	1104.345
	246.274
	1132.170
	589.010

Caspase-1 1st collection

Favorable	Unfavorable
1.478	2.052
0.970	1.544
2.567	2.960
17.892	1.733
	0.918
	4.818
	7.260
	7.985
	14.771
	14.031
	14.411
	1.158
	20.554
	5.647

Caspase-1 2nd collection

Favorable	Unfavorable
1.945	3.328
2.119	2.522
2.637	1.588
1.486	1.540
	3.339
	2.062
	5.962
	2.422
	2.266
	4.123
	9.434

	1.588
	6.516
	4.639

<u>Caspase-1 4th collection</u>

Favorable	Unfavorable
2.583	4.649
2.731	3.455
2.016	1.836
4.145	3.989
	5.130
	5.148
	3.904
	5.165
	2.570
	4.065
	2.892
	8.889
	6.015
	4.770

<u>Serum IL-18 1st collection</u>

Favorable	Unfavorable
233.355	318.533
43.723	104.232
130.457	47.804
160.849	104.986
306.775	119.067
	77.442
	167.342
	304.151
	213.145
	252.959
	178.019
	364.816
	190.116
	111.408
	125.183
	273.831

<u>Serum IL-18 2nd collection</u>

Favorable	Unfavorable
259.711	221.660
119.592	95.383

146.893	46.586
192.685	228.044
246.165	237.482
	224.073
	120.714
	384.541
	171.978
	347.315
	215.263
	390.554
	189.629
	284.101
	233.669
	369.261

Serum IL-18 4th collection

Favorable	Unfavorable
335.670	260.689
145.726	123.252
70.752	130.532
212.354	213.299
164.056	227.538
	210.336
	168.783
	293.650
	193.186
	377.697
	230.724
	612.271
	207.186
	297.998
	88.031
	423.688
